# Prevalence of sexual coercion and associated factors among adolescents and young adults in Africa: a systematic review and meta-analysis

**DOI:** 10.3389/frph.2025.1697868

**Published:** 2025-11-28

**Authors:** Posi Emmanuel Aduroja, Adebukunola Olajumoke Afolabi, Ayobami Adebayo Bakare, Oluwaseyi Isaiah Olabisi, Atimi Atinga, Hameed Akande Bashiru, Abiola Solomon, Iyanu Adufe, Oziegbe Oghide, Oluchukwu Perpetual Okeke, Olunike Rebecca Abodunrin, Folahanmi Akinsolu, Olajide Odunayo Sobande

**Affiliations:** 1 Department of Public Health, Faculty of Basic Medical and Health Sciences, Lead City University, Ibadan, Oyo, Nigeria; 2 Department of Nursing Science, Faculty of Basic Medical Sciences, College of Health Sciences, Obafemi Awolowo University, Ile-Ife, Nigeria; 3 Department of Global Public Health, Karolinska Institutet, Stockholm, Sweden; 4 Department of Community Medicine, University College Hospital, Ibadan, Oyo, Nigeria; 5 Department of Mental Health and Psychiatric Nursing, Faculty of Nursing Sciences, College of Health Science, Bowen University, Iwo, Nigeria; 6 Department of Public Health Technology, Taraba State College of Health Technology, Taraba, Nigeria; 7 Department of Animal Sciences, Obafemi Awolowo University, Ile-Ife, Nigeria; 8 Department of Medical Biochemistry, Eko University of Medicine and Health Sciences, Lagos, Nigeria; 9 Department of Public Health, Faculty of Basic Medical Sciences, Osun State University, Osun, Nigeria; 10 Accident & Emergency Department, Lagos University Teaching Hospital, Lagos, Nigeria; 11 Nigerian Institute of Medical Research Foundation, Yaba, Lagos, Nigeria; 12 Department of Epidemiology and Biostatistics, Nanjing Medical University, Nanjing, China; 13 Center for Reproduction and Population Studies, Clinical Sciences Department, Nigerian Institute of Medical Research, Yaba, Lagos, Nigeria

**Keywords:** sexual violence, sexual abuse, young people, teenager, gender-based violence, meta-analysis, sub-Sahara Africa

## Abstract

**Introduction:**

Sexual coercion is a major public health and human rights concern, yet its burden among African adolescents and young adults remains poorly synthesized. This review aimed to estimate the prevalence of sexual coercion in this population and examine variations by gender, setting, and region.

**Methods:**

We systematically searched Web of Science, Scopus, MEDLINE/PubMed, and CINAHL for studies published between January 2000 and June 2025. Two reviewers independently screened, extracted, and appraised eligible studies. A narrative synthesis was integrated with a random-effects meta-analysis due to anticipated high heterogeneity to derive pooled prevalence estimates and conduct subgroup analyses.

**Results:**

Thirty-three studies involving 63,934 participants from 14 African countries were included. The pooled prevalence of sexual coercion was 20% (95% CI: 17%–23%). Females reported higher prevalence (23%) than males (19%). School-based studies showed greater prevalence (26%) compared with community-based studies (16%).

**Discussion:**

Sexual coercion remains a major public health and human rights concern among African adolescents, particularly school-going females. Strengthening school- and community-based programs that integrate comprehensive sexuality education on consent, negotiation, and gender equality is essential. Developing a standardized, context-sensitive tool for measuring sexual coercion will also enhance evidence quality and policy response.

**Systematic Review Registration:**

https://www.crd.york.ac.uk/PROSPERO/view/CRD420251067378, PROSPERO CRD420251067378.

## Introduction

Sexual coercion is a pervasive yet under-examined form of gender-based violence, disproportionately affecting adolescents and young adults (10–24 years old) across diverse relational contexts. Globally, prevalence estimates vary widely, a global meta-analysis estimated coercive sex at 11.4% among women and 6.8% among men, with other studies reporting 33% in Bosnia and Herzegovina and Croatia, 26.7% in Germany, and consistently high rates across several African countries (1–6). For instance, 68% of in-school youth in Ghana (5), 45% of South African adolescents, predominantly aged 13–14 years (4), and more than one-third of Nigerian university students (3) have reported experiencing sexual coercion. These figures underscore the magnitude of the problem yet also highlight measurement heterogeneity in African studies, which include inconsistency in item wording and conflating coercion to bigger constructs of sexual violence and a lack of research that focused specifically on the phenomenon of coercion.

Conceptually, sexual coercion encompasses a broad spectrum of non-consensual sexual encounters. Early definitions of sexual coercion emphasized on overt forms of force—whether verbal, cultural, economic, or physical, used to compel individuals into sexual activity (7). Later work expanded the definition to include more subtle pressures, such as the use of alcohol and drugs, emotional manipulation, and persistent refusal to respect boundaries (8). Contemporary perspectives recognize that coercion may also occur through threats, deception, false promises, or abuse of authority and power, particularly against vulnerable populations (9, 10). Today, sexual coercion is increasingly understood as the use of nonphysical means to obtain sexual contact (i.e., fondling, oral sex, or intercourse) with a non-consenting female partner (11). These strategies may involve lying, guilt, making false promises, arguing constantly, intoxication, threatening to break up, or disobeying the victims’ vocal demands to stop (without resorting to physical force) (12).

The health and social consequences of sexual coercion are profound, as victims face elevated risks of unintended pregnancies, sexually transmitted infections (including HIV), mental health disorders such as depression and anxiety, as well as academic underperformance (13–16). Moreover, coerced individuals are more likely to engage in risky sexual behaviors, including early sexual debut, multiple partnerships, transactional sex, and inconsistent condom use (14). These consequences reinforce a cycle of vulnerability, particularly in resource-constrained settings.

In Africa, adolescents and young adults (ages 10–24) face heightened exposure due to early sexual initiation, entrenched gender inequalities, harmful social norms, poverty, and limited access to comprehensive sexuality education (17). Such conditions create environments conducive to manipulation and exploitation. Yet, despite growing recognition of the problem, prevalence estimates across the continent remain fragmented and inconsistently measured. Existing evidence is largely derived from country-specific surveys or narrow subgroups (e.g., school-attending youth, clinic attendees), limiting the ability to generate continent-wide insights.

Although rape and physical sexual assault have been well-known in the literature and policy-making processes, sexual coercion, especially non-physical means (including verbal coercion, emotional control, or relationship penalty threats), is a relatively understudied topic despite being a significant portion of unwanted sexual experiences. Research has repeatedly shown that coerced sex is frequently absent of overt force and is therefore not well-reported and categorized as sexual violence within standard prevalence rates, thus concealing its true burden (18). Furthermore, the psychological and relational damages of sexual coercion are shown to be potentially similar to those caused by physical assault, which explains that harm can be predetermined not solely by the physical force (18). This evidence gap poses major challenges for research, programming, and policy. Without consolidated data, it is difficult to quantify the true burden of sexual coercion within Africa's diverse contexts.

A systematic review and meta-analysis can address this gap by synthesizing available prevalence data, identifying demographic and contextual patterns, and highlighting methodological limitations in existing studies. A narrow analysis of sexual coercion as a unique phenomenon is necessary to produce nuanced evidence, to enhance the level of measurement accuracy, and to create interventions that address less and less obvious manifestations of sexual violation. This systematic review and meta-analysis aim to address this gap by summarizing existing literature on sexual coercion as an independent construct and not when it is subsumed within rape or general sexual violence, and importantly, evidence generated herein is crucial for guiding policies and programs aligned with the Sustainable Development Goals (SDGs), notably Goal 5 (gender equality) and Goal 3 (good health and well-being).

Accordingly, this review therefore aims to to estimate the prevalence of sexual coercion in this population and explore examine variations by gender, setting, and region. By consolidating fragmented findings, this study seeks to advance a more comprehensive and contextually grounded understanding of sexual coercion, thereby informing culturally sensitive, rights-based strategies to safeguard the sexual and reproductive health of young people across Africa.

## Methods

### Protocol and registration

The review protocol was registered with the International Prospective Register of Systematic Reviews (PROSPERO ID = CRD420251067378). The review was designed, conducted and reported in accordance with the Preferred Reporting Items for Systematic Reviews and Meta-Analyses (PRISMA 2020) guidelines (See Supplementary Appendix A for the PRISMA Checklist).

### Research questions

This review aims to answer the following questions:

1. What is the prevalence of sexual coercion among adolescents and young adults (10–24 years) in Africa?
2. What factors are associated with sexual coercion among adolescents and young adults (10–24 years) in Africa?

### Search strategy

We conducted a systematic and comprehensive search across four major electronic databases selected for their relevance to the topic: Web of Science (Thomson Reuters), Scopus (Elsevier), MEDLINE/PubMed, and CINAHL (EBSCOhost). The search combined controlled vocabulary (e.g., MeSH terms) and free-text keywords, using Boolean operators. The core search string was:

(Prevalence (MeSH) OR prevalence OR frequenc* OR occurrence* OR pattern*) AND (“Sexual coercion” OR “sexual violence” OR “sexual assault” OR “sexual abuse” OR “non-consensual sex” OR “sexual manipulation” OR “sexual exploitation”) AND (Adolescent (MeSH) OR Adolescen* OR youth* OR Teen* OR “young people” OR “young adult*”) AND (Algeria OR Angola OR Benin OR Botswana OR “Burkina Faso” OR Burundi OR Cameroon OR “Cape Verde” OR “Central African Republic” OR Chad OR Comoros OR Congo OR “Democratic Republic of the Congo” OR Djibouti OR Egypt OR “Equatorial Guinea” OR Eritrea OR Eswatini OR Ethiopia OR Gabon OR Gambia OR Ghana OR Guinea OR “Guinea-Bissau” OR Ivory Coast OR “Côte d’Ivoire” OR Kenya OR Lesotho OR Liberia OR Libya OR Madagascar OR Malawi OR Mali OR Mauritania OR Mauritius OR Morocco OR Mozambique OR Namibia OR Niger OR Nigeria OR Rwanda OR “São Tomé and Príncipe” OR Senegal OR Seychelles OR “Sierra Leone” OR Somalia OR “South Africa” OR “South Sudan” OR Sudan OR Tanzania OR Togo OR Tunisia OR Uganda OR Zambia OR Zimbabwe).

The search was limited to studies published between January 1, 2000, and June 30, 2025, to capture contemporary and contextually relevant evidence. In addition, Google Scholar was searched to identify potentially relevant studies not indexed in the selected databases.

No restrictions were applied on language during the initial search phase, and no automatic filters were used. Full search strategies for each database, including field tags and Boolean logic, are provided in Supplementary Appendix B to ensure replicability.

### Eligibility criteria

We included observational studies, specifically cross-sectional surveys and baseline data from cohort studies (only data collected before any intervention or exposure classification were used to minimize selection bias) that reported the prevalence of sexual coercion among adolescents and young adults aged 10–24 years living in African countries. Studies were eligible if data were disaggregated for this age group or could be extracted separately. Only articles published between January 2000 and June 2025 were considered.

We excluded qualitative studies, intervention trials, case series, case reports, reviews, editorials, and commentaries. Studies conducted outside Africa or those that did not report prevalence estimates of sexual coercion were also excluded.

A detailed summary of the inclusion and exclusion criteria is provided in Supplementary Appendix C.

### Study selection process

Study selection was guided by the PEOS framework (19). The population of interest was adolescents and young adults aged 10–24 years residing in Africa. The primary exposure was sexual coercion, and the outcome of interest was its prevalence and associated factors.

All identified records were imported into Rayyan software, for systematic review screening (20). Rayyan's automated deduplication feature was used to remove duplicate records.

Following deduplication, two reviewers (AOA and AAB) independently screened titles and abstracts against the predefined eligibility criteria. Articles that did not meet the inclusion criteria were excluded, and reasons for exclusion were documented (Supplementary Appendix C). Eligible studies were then subjected to full-text review by two independent reviewers (PEA and OIO). Discrepancies at both stages were resolved through discussion, and when consensus could not be reached, a third reviewer (FTA) was consulted to arbitrate.

### Data collection process

Data extraction was carried out on all studies that met the eligibility criteria. We focused on key characteristics relevant to the review question, including study identifiers (authors, year, country, and region), participant demographics (age range, sex, setting), study design, measurement of sexual coercion, prevalence estimates (overall and sex-disaggregated where available), and associated factors.

To make the studies with a high level of methodological heterogeneity consistent, all contextual variables were standardised and coded in a systematic way. Specifically, gender categories were standardised through recoding the reported categories that were put under the mutually exclusive male, female or both male and female regardless of the terms that were actually used. The setting of the study was divided into school-based, community-based, facility-based (e.g., clinic, hospital), and DHS. Standardisation of geographic region was based on the United Nations sub regional grouping (e.g., Eastern Africa, Western Africa, Southern Africa) whilst the names of individual countries have been retained to preserve the granularity. The multinational studies that included more than one country were coded as multinational and each constituent country was set separately in the synthesis, provided that it had stratified data.

Raw categories that were not directly comparable were recoded into single brackets (e.g., reference to adolescents was expressed 10–19, 10–24 as adolescents or 19–24 years as young adults), but the original categories were also kept in a memo column to provide traceability. Similarly, the inconsistencies in the operational definitions of sexual coercion were also reported in the original form and in the way they were transferred to the wider categories of analysis.

A standardized extraction sheet was developed, pilot-tested on a subset of eligible studies, and refined for clarity and completeness. The final version of the extraction sheet is provided in Supplementary Appendix D to ensure transparency and replicability.

Data extraction was performed independently by two reviewers (AOA and PEA) following a protocol consistent with PRISMA 2020 guidelines. The process was guided by the PEOS framework (Table 1) to ensure consistent capture of population, exposure, outcomes, and study design elements. To minimize bias, reviewers were trained on the use of the extraction tool before the process began.

**Table 1:** Characteristics of included studies on the prevalence of sexual coercion.

Author(s)	Country	Study setting	Age	sample size	Prevalence of sexual coercion			Risk of bias
					Overall	Male	Female	
Aduayi et al. (21)	Nigeria	DHS	15–24	12,626	132	NR	NR	High
Amo-Adjei et al. (22)	Ghana	Community	13–24	1,272	120	28	92	Low
Bekele et al. (23)	Ethiopia	school	12–24	764	192	NR	NR	Low
Beyene et al. (24)	Ethiopia	school	15–24	1,064	258	NR	NR	Low
Chime et al. (25)	Nigeria	School	11–19	325	116	NR	NR	Medium
Decraen et al. (26)	Rwanda	School	19–23	285	16	3	13	Medium
Erulkar (27)	Kenya	Community	10–24	1,753	127	35	92	High
Fan et al. (28)	Malawi	community	13–24	1,029	329	NR	NR	Low
Garoma et al. (29)	Ethiopia	community	10–24	641	167	NR	NR	Low
Garoma Abeya (30)	Ethiopia	community	10–24	632	233	NR	NR	Low
Goessmann et al. (31)	Tanzania & Uganda	school	12–17	1,402	114	73	41	Low
Haile et al. (32)	Ethiopia	School	18–19	830	77	NR	NR	Low
Landis et al. (33)	Congo	Community	13–14	350	30	NR	NR	Medium
Maharaj and Munthree (34)	South Africa	community	14–24	1,130	520	NR	NR	Medium
Meinck et al. (35)	South Africa	community	10–17	3,401	125	NR	NR	Low
Mlyakado and Li (36)	Tanzania	School	13–17	1,116	234	NR	NR	Medium
Moore et al. (37)	Burkina Faso	community	12–19	2,908	142	NR	NR	Low
Moore et al. (37)	Ghana	community	12–19	2,157	265	NR	NR	Low
Moore et al. (37)	Malawi	community	12–19	2,027	154	NR	NR	Low
Moore et al. (37)	Uganda	community	12–19	2,488	97	NR	NR	Low
Naidoo et al. (38)	South Africa	school		434	54	NR	NR	Low
Nguyen et al. (39)	Uganda	community	13–24	5,804	621	NR	NR	Low
Nguyen et al. (39)	Zambia	community	13–24	1,819	291	NR	NR	Low
Nguyen et al. (39)	Nigeria	community	13–24	4,203	588	NR	NR	Low
Odeyemi et al. (40)	Nigeria	Community	10–19	350	10	NR	NR	Medium
Owusu-Addo et al. (41)	Ghana	community	13–19	853	30	NR	NR	Low
Perry et al. (42)	Uganda	Community		1,126	274	25	122	Low
Richter et al. (4)	South Africa	Community	11–18	1,882	385	NR	NR	Low
Rizo et al. (43)	Ghana	school	16–18	103	47	NR	NR	Low
Rumble et al. (44)	Zimbabwe	Community	18–24	1,156	50	8	42	Low
Seidu et al. (5)	Ghana	Schools	10–24	979	672	408	264	Medium
Stark et al. (45)	DRC	Community	13–19	377	62	NR	NR	Low
Stark et al. (45)	Ethiopia	Community	13–19	919	251	NR	NR	Low
Swedo et al. (46)	Malawi	community	13–24	595	231	NR	NR	Low
Tenkorang et al. (47)	Ghana	school	13–17	726	176	78	97	Low
Tusiime et al. (48)	Uganda	facility-based	15–24	416	100	NR	NR	Low
Worku and Addisie (49)	Ethiopia	school	12–21	216	17	NR	NR	High
Ybarra et al. (50)	Uganda	School	12–19	354	311	96	35	Medium
Zablotska et al. (51)	Uganda	community	15–24	3,422	458	NR	NR	Low

Discrepancies between the two reviewers were resolved through discussion. Where disagreements persisted, a third reviewer (ORA) was consulted for arbitration, and consensus was reached. This multi-reviewer approach reflects best practices in systematic reviews, enhancing the reliability and accuracy of extracted data.

### Data synthesis

Given the substantial heterogeneity across study designs, populations, settings, and definitions of sexual coercion, a narrative synthesis was first undertaken to describe and compare findings across included studies. This allowed us to contextualize prevalence estimates and associated factors within their methodological and geographical settings.

Key characteristics of each study were systematically tabulated to provide an overview of the evidence base. Extracted variables included: study ID, title, author(s), year of publication, country, African sub-region, study design, data collection instrument, study setting (e.g., school, community, health facility), target population, age distribution, sample size (total and sex-disaggregated), prevalence estimates (overall and, where reported, sex-specific), associated factors.

In addition to narrative synthesis, quantitative pooling was conducted using meta-analysis for prevalence estimates and selected associated factors (see Statistical Analysis section). Subgroup analyses were planned *a priori* to examine differences by sex, type of coercion, study setting, sample size, and region. Findings from both the narrative and quantitative syntheses were integrated in the result section to provide a comprehensive understanding of sexual coercion among adolescents and young adults in Africa.

### Risk of bias assessment

The quality of included studies were assessed using the Appraisal Tool for Cross-Sectional Studies (AXIS) (52), which evaluates 20 items related to study design, sampling, measurement reliability, ethical compliance, and clarity of reporting. Two reviewers independently (AAB and OIO)applied the AXIS tool to each study. The overall quality of each study was summarized narratively and used to inform the interpretation of findings.

### Statistical analysis

We conducted a meta-analysis of prevalence estimates using a random-effects model (DerSimonian–Laird method), anticipating variation in true effect sizes due to differences in study populations, contexts, and definitions of sexual coercion. The random-effects model was therefore considered appropriate because it accounts for both within-study sampling error and between-study heterogeneity, providing estimates that are more generalizable across diverse settings.

To address potential variance instability, particularly in studies with small sample sizes or very high or very low prevalence values, the Freeman–Tukey double arcsine transformation was applied to all proportion estimates prior to pooling. This transformation reduces the risk of skewness and prevents undue influence of studies at the extremes. The pooled estimates were then back-transformed to original proportions to facilitate interpretation.

Pooled prevalence values with corresponding 95% confidence intervals (CIs) were reported. Statistical heterogeneity was evaluated using Cochran's *Q* test and quantified using the *I*^2^ statistic, with values of 25%, 50%, and 75% interpreted as low, moderate, and high heterogeneity, respectively. Between-study variance (*τ*^2^) was also calculated to capture the extent of absolute heterogeneity. A random-effects model was used to account for methodological and contextual heterogeneity across included studies. Given that there should be heterogeneity in population, measures, conceptualisations of sexual coercion, and the research context, it was postulated that the true prevalence parameter could vary and not be equal to a common effect size; thus, a fixed effect model was deemed inappropriate. The random-effects model enabled a more cautious and generalizable pooled estimate, as it combined the within-study and between-study variance. To further investigate sources of heterogeneity, we conducted a series of pre-specified subgroup analyses. These were stratified by gender, age group, type of coercion (penetrative vs. non-penetrative), study setting (school-based vs. community-based), sample size, and geographic region. We also performed sensitivity analyses by sequentially removing each study (leave-one-out analysis) to assess the robustness of the pooled estimates and identify any single study with undue influence.

Finally, publication bias was assessed both visually and statistically. Funnel plots were examined for asymmetry, and Egger's regression test was applied to formally test for small-study effects. In instances where evidence of bias was detected, the trim-and-fill method was used to estimate the number of potentially missing studies and to adjust the pooled prevalence accordingly.

### Ethics

Ethical approval and informed consent were not required for this study as it is a systematic review and meta-analysis of previously published studies. No primary data were collected, and there was no direct involvement of human participants. All data included in this review were obtained from publicly available, peer-reviewed articles indexed in scientific databases.

## Results

**Figure 1:**
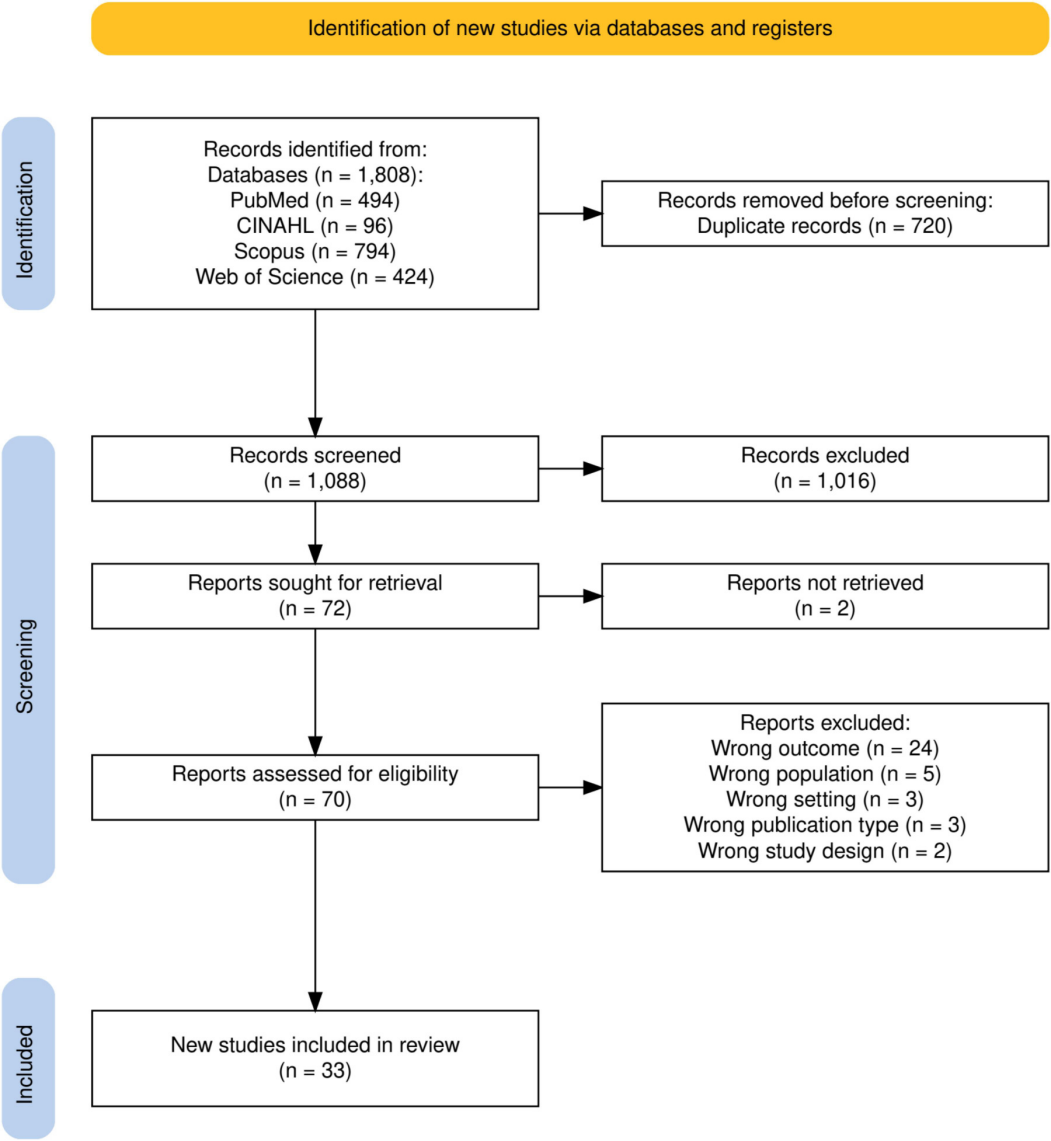
PRISMA flow chart.

A total of 1,808 records were obtained from the systematic database searches, which was carried out on June 30, 2025. Duplicate entries were automatically detected and removed using the Rayyan software. After removing 720 duplicates, 1,088 records were screened by title and abstract, 1,016 papers did not meet our eligibility criteria and these were excluded. Full-text assessment was conducted for remaining 72 studies, and 39 were excluded for reasons which are detailed in Supplementary Appendix E. Ultimately, 33 studies were included in the final review (4, 5, 21–33, 35–51, 53). The study selection process is illustrated in the PRISMA 2020 flow diagram (Figure 1).

### Characteristics of included studies

The characteristics of studies included in this review are described in Table 1. The studies were published between 2002 (49) and 2025 (22). Seven were conducted in Ghana (5, 22, 37, 41, 43, 47), seven in Uganda (31, 37, 39, 42, 48, 50, 51), seven in Ethiopia (23, 24, 29, 30, 32, 49), four in Nigeria (21, 25, 39, 40), four in South Africa (4, 34, 35, 38), three in Malawi (28, 37, 46), two in Tanzania (31, 36), and one each in Congo (33), Democratic Republic of Congo (45), Rwanda (26), Zimbabwe (44), Kenya (27), Zambia (39), and Burkina Faso (37). Most studies were community-based (*n* = 20), while 13 were school-based, two were facility-based, and one used DHS data (21). Sample sizes ranged from 103 (43) to 12,626 (21). The participants’ age ranged from 10 to 24 years. The prevalence of sexual coercion varied widely, from as low as 3% (40) to 69% (5).

### Risk of bias and quality of individual studies

The methodological quality of included studies was assessed using the Appraisal Tool for Cross-Sectional Studies (AXIS) (52), which evaluates 20 items related to study design, sampling, measurement reliability, ethical compliance, and clarity of reporting (Supplementary Appendix F). Most studies clearly stated their aims and objectives, used appropriate study designs, and provided sufficient details of study population and study settings. Twenty-one studies were assessed to have low risk of bias (4, 22–24, 28, 29, 31, 32, 35, 37–39, 41–48, 51), nine studies were assessed to have medium risk of bias (5, 25, 26, 30, 33, 34, 36, 40, 50). Only three were assessed to have a high risk of bias (21, 27, 49) and hence excluded from meta-analysis.

### Meta-analysis of prevalence

**Figure 2:**
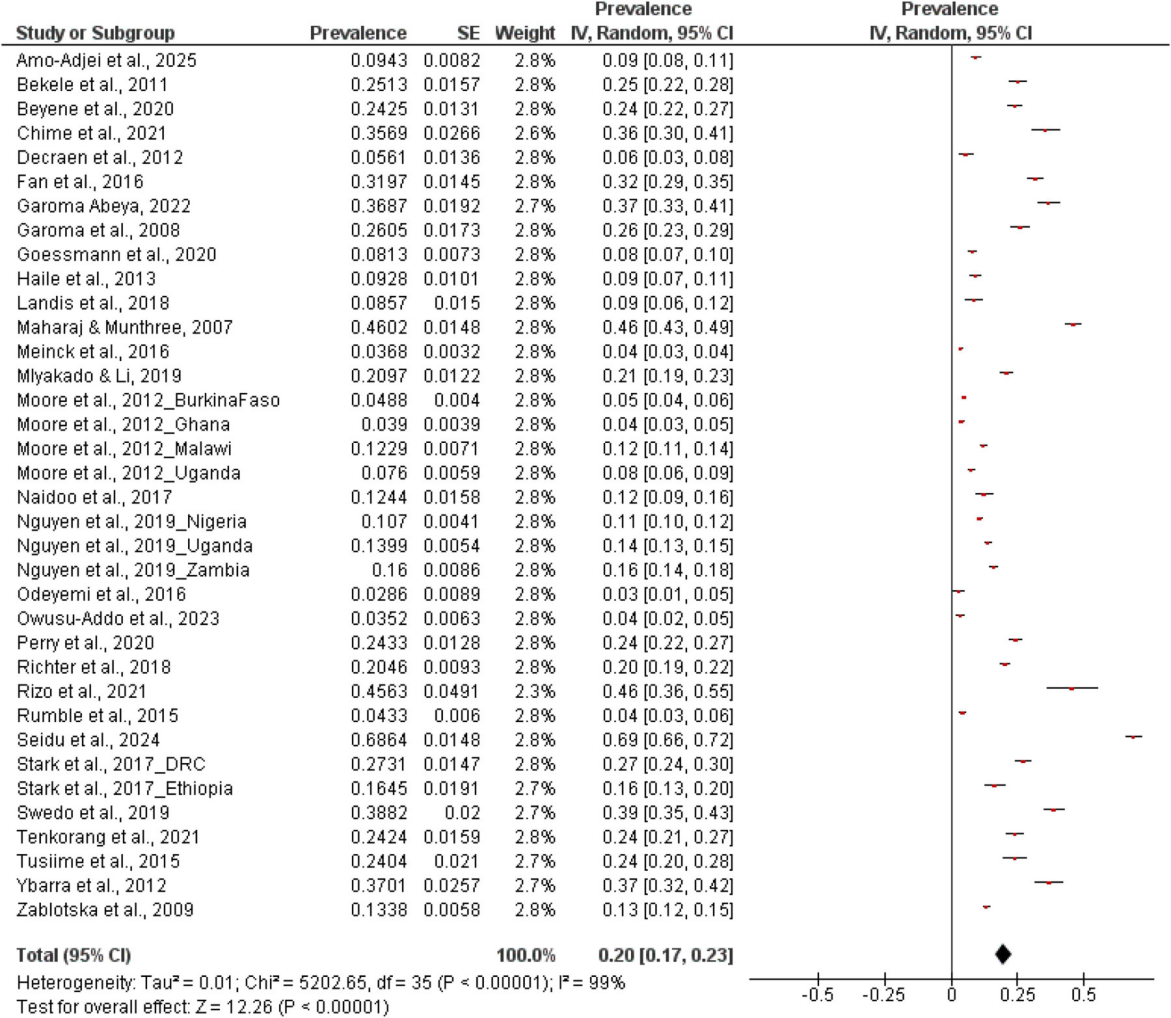
Pooled prevalence of sexual coercion.

A total of 33 studies were included in the quantitative synthesis (4, 5, 21–28, 30–37, 39, 40, 43, 45, 48, 50, 51, 54). Using a random-effect model, the pooled prevalence of sexual coercion across all population was estimated at 0.20 (95% CI: 0.17–0.23) (Figure 2). Heterogeneity was very high (*I*^2^ = 99%, *τ*^2^ = 0.01, *χ*^2^ = 5,202.65, *p* < 0.001), indicating substantial variability beyond chance. Reported prevalence ranged widely, from as low as 3% (40) to as high as 69% (5), with several studies reporting moderate to high levels such as 46% (34), 39% (46), 36% (25), and 32% (28). When stratified by sex, eight studies provided male-specific data, yielding a pooled prevalence of 19% (95% CI: 0.08–0.30). Heterogeneity was again high (*I*^2^ = 100%, *τ*^2^ = 0.02, *χ*^2^ = 1,424.55, *p* < 0.01), with prevalence estimates ranging from 1% (44) to 70% (5). For females, the pooled prevalence across eight studies was higher at 23% (95% CI: 0.12–0.33). Heterogeneity remained substantial (*I*^2^ = 99%, *τ*^2^ = 0.02, *χ*^2^ = 682.73, *p* < 0.01), with study-specific ranging from 6% (31) to 67% (5).

**Figure 3:**
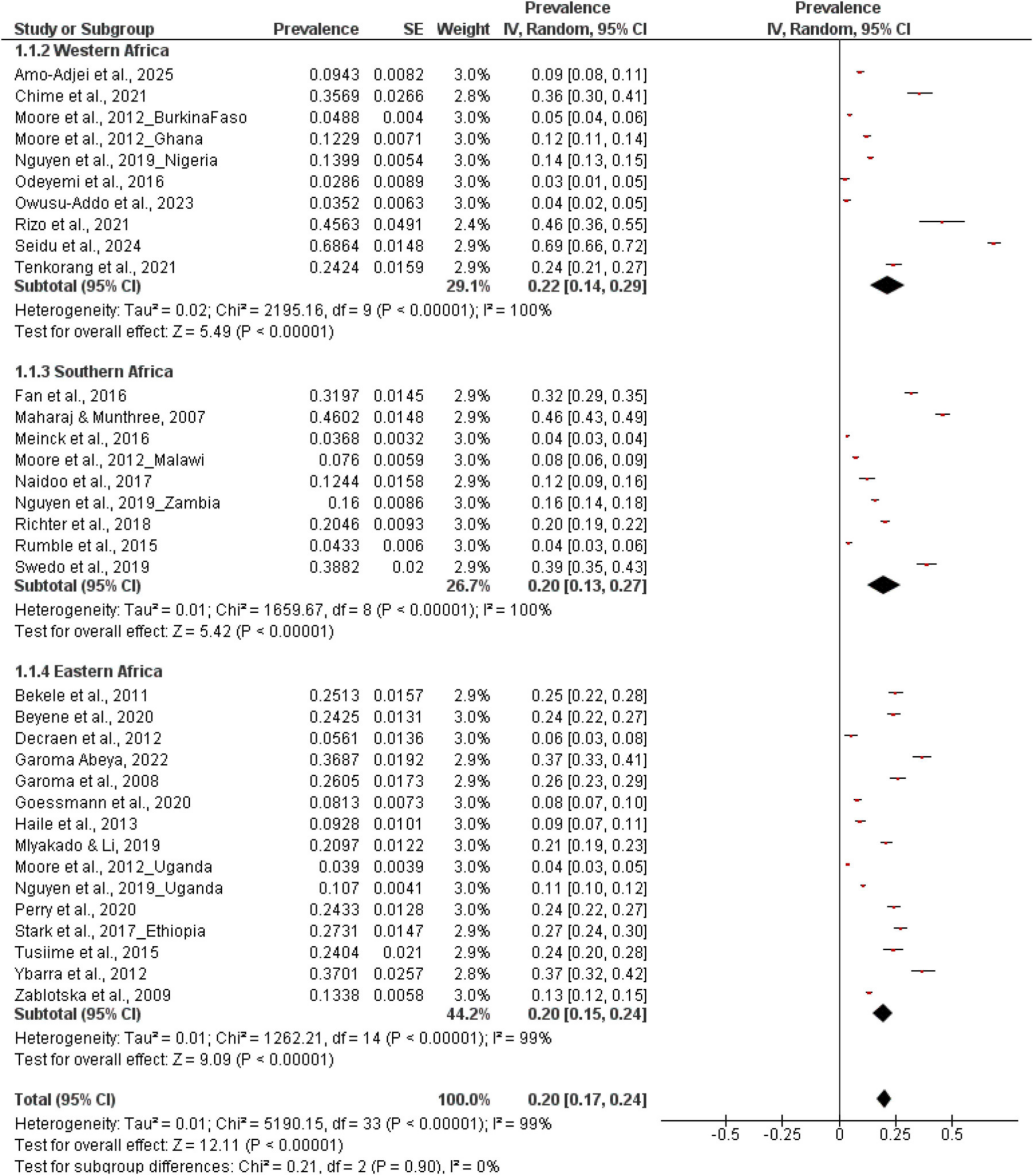
Prevalence of sexual coercion by regions.

Regionally, the highest prevalence of sexual coercion was reported in West Africa at 22% (95% CI: 0.14–0.29; *n* = 10), with both East Africa at 20% (95% CI: 0.15–0.24; *n* = 15), and Southern Africa at 20% (95% CI: 0.13–0.29; *n* = 9) (Figure 3).

Patterns also varied by study size, region, and setting. Prevalence was inversely related to sample size, with studies with fewer than 1,000 participants reporting higher estimates at 25% (95% CI: 0.17–0.33; *n* = 17) for samples exceeding 1,000 participants, prevalence estimate was lower at 15% (95% CI: 0.12–0.18; *n* = 13). Finally, setting-specific analyses showed that school-based studies (*n* = 12) reported higher prevalence at 26% (95% CI: 0.16–0.36) than community-based studies (*n* = 17) at 16% (95% CI: 0.13–0.20). Summary of prevalence of sexual coercion in all subgroups is presented in Table 2.

**Table 2:** Prevalence of the sexual coercion in all the subgroups.

Variable	Category	No. of studies	Prevalence [95% CI]	*I* ^2^	*τ* ^2^	*χ* ^2^	*p*
Age	Adolescents	19	0.16 [0.13, 0.19]	99%	0.00	1,494.30	<0.001
	Adolescents & Young Adults	15	0.26 [0.20, 0.32]	99%	0.01	2,474.72	<0.001
Gender	Female	21	0.23 [0.17, 0.29]	99%	0.02	2,256.59	<0.001
	Male	15	0.16 [0.11, 0.20]	99%	0.01	1,967.90	<0.001
Region	Western Africa	10	0.22 [0.14, 0.29]	100%	0.02	2,195.16	<0.001
	Southern Africa	9	0.20 [0.13, 0.27]	100%	0.01	1,659.67	<0.001
	Eastern Africa	15	0.20 [0.15, 0.24]	99%	0.01	1,262.21	<0.001
Sample Size	Less than 1,000	18	0.25 [0.17, 0.33]	99%	0.03	2,579.92	<0.001
	More than 1,000	18	0.15 [0.12, 0.18]	99%	0.00	2,271.80	<0.001
Setting	School-based	12	0.26 [0.16, 0.36]	99%	0.03	1,715.55	<0.001
	Community-based	23	0.16 [0.13, 0.20]	99%	0.01	2,860.63	<0.001

### Meta-Analysis of factors associated with sexual coercion among adolescents and young adults

**Figure 4:**
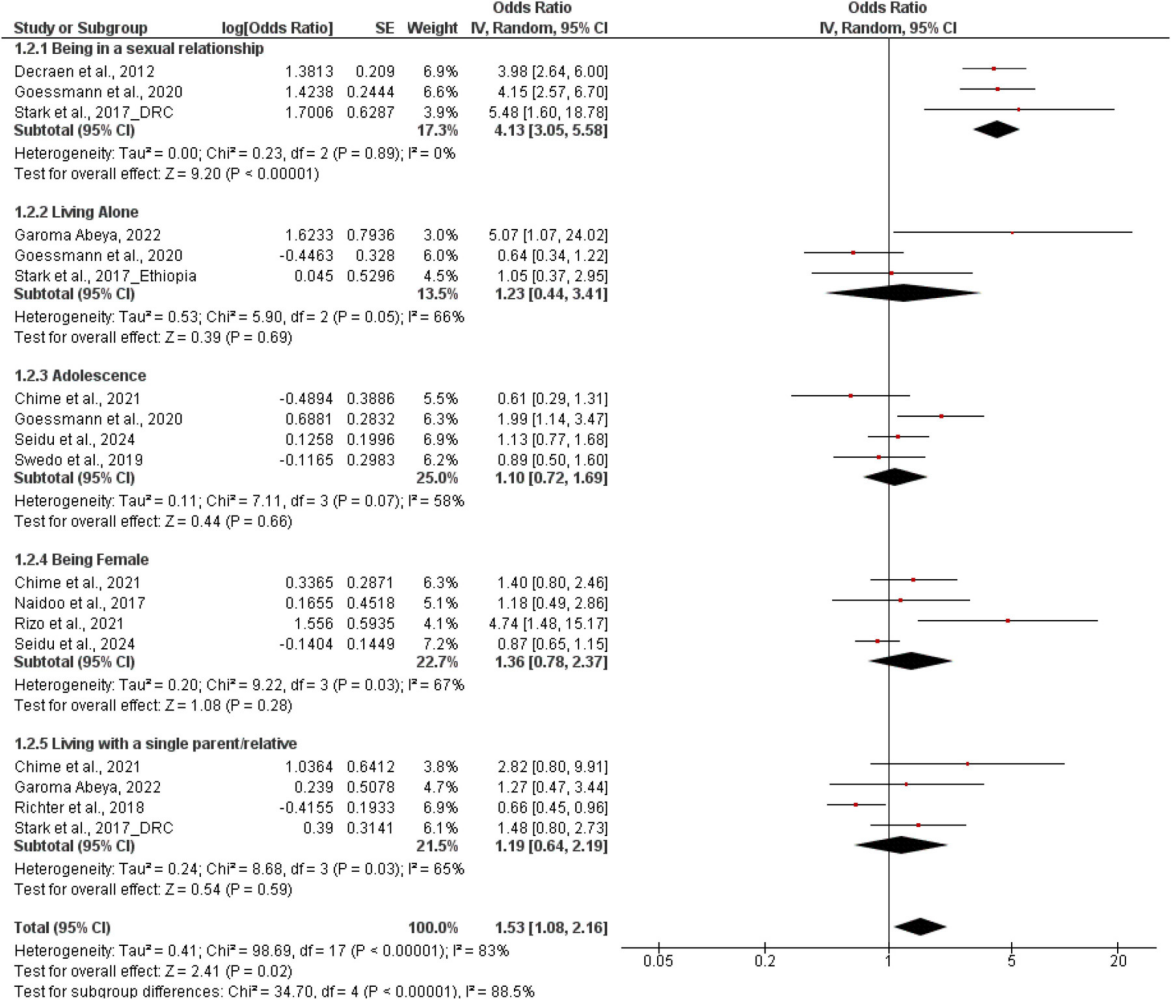
Pooled effect size of associated factors for sexual coercion.

The meta-analysis investigated the variables that were related to sexual coercion in the adolescents and young adults in Africa, through the application of a random-effects model. In general, the participants who were exposed to the considered factors were 1.53 times more likely to be sexually coerced than not exposed to them (OR = 1.53; 95% CI: 1.08–2.16; *p* = 0.02). Nonetheless, the heterogeneity was significant among the studies (*τ*^2^ = 0.41; *χ*^2^ = 98.69, df = 16; *p* < 0.00001; *I*^2^ = 83%), suggesting much heterogeneity in the results of different studies. Of the subgroups, the only one that was found to be statistically significant to relate to sexual coercion (OR = 4.13; 95% CI: 3.05–5.58; *p* < 0.00001) was the being in a sexual relationship, with no heterogeneity observed (*I*^2^ = 0%). Sexual coercion was also not significantly correlated with other variables such as living alone (OR = 1.23; 95% CI: 0.44–3.41; *p* = 0.69), adolescence (OR = 1.10; 95% CI: 0.72–1.69; *p* = 0.66), being female (OR = 1.36; 95% CI: 0.78–2.37; *p* = 0.28), and living with a single parent or relative (OR = 1.19; 95% CI: 0.64–2.19; *p* = 0.59). These results underscore the fact that although other social and demographic factors might be relevant, sexual relationship involvement has been the most predictable and prominent factor of sexual coercion risk among the adolescents and young adults, in the African context (Figure 4).

### Publication bias

**Figure 5:**
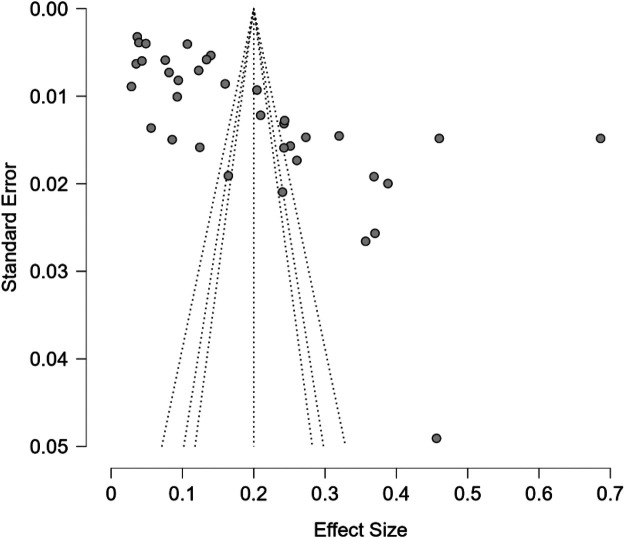
Funnel plot of publication bias based on Egger's regression test for sexual coercion.

The meta-analysis was analyzed to determine the extent of publication bias using the Egger test and trim and fill technique where the findings showed possible asymmetry and adjustment of the overall effect estimate. The regression test of funnel plot asymmetry conducted by Egger gave a statistically significant value, t value = 6.097, df = 34, and *p* = .001. This means that the funnel plot is very asymmetrical indicating that the meta-analysis could have publication bias or a small-study effect. The trim and fill technique gave 0 missing studies on the left hand side of the funnel plot which implies no apparent gap because of suppressed negative results. Nevertheless, the adjusted trim and filled effect estimate was 0.149 (95% CI: 0.121–0.196, *p* < 0.001), which was lower than the initial unadjusted effect estimate of 0.200 (95% CI: 0.151–0.249, *p* < 0.001). The downward adjustment implies that the original pooled estimate might have been overstated probably because of the effect that the larger or more extreme impacts of smaller studies had (Figure 5).

### Sensitivity analysis

The leave-one-out analysis shows that excluding any single study does not substantially alter the overall proportion of sexual coercion, indicating that the meta-analytic estimate is stable and not unduly influenced by any individual study (Supplementary Appendix G).

## Discussion

This systematic review and meta-analysis estimated the prevalence of sexual coercion and examined its associated factors among adolescents and young adults in Africa. Overall, approximately one in every five adolescents and young adults reported having experienced sexual coercion, with prevalence significantly higher among females. This gender disparity is consistent with global evidence showing that women and girls disproportionately experience sexual and gender-based violence (54, 55). These disparities are evidence of a well ingrained gender and structural inequalities that determine vulnerability. Female adolescents and young adults tend to be forced into unequal power dynamics both in intimate relations and in institutions when cultural norms can condone the dominance of men or the silence of victims (57). In schools, interplay of hierarchy of power, financial reliance and lack of sexual autonomy elevates the chances of being sexually coerced by peers, teachers or older mates (5). Moreover, the stigma of sexual violence can provoke the underreporting of the male population, which may further increase the apparent gender gaps in prevalence statistics (57).

The geographical distribution of studies revealed important gaps. Most research originated from East and West Africa, with fewer studies from Southern Africa, only one from Central Africa, and none from North Africa. Underrepresentation of certain regions likely reflects cultural sensitivities, limited funding, and socio-political constraints that hinder open discussion of adolescent sexuality. This uneven evidence obscures the true continental burden and undermines equitable sexual and reproductive health programming. Strengthening research capacity and encouraging culturally sensitive, regionally inclusive studies are therefore essential. Moreover, the relatively higher prevalence reported in West Africa suggests that interventions must be adapted to local contexts to be effective.

Subgroup analyses further revealed differences by age, study setting, and sample size. While adolescents reported lower prevalence than an expanded population of both adolescents and young adults, it suggests greater vulnerability during early stages of sexual exploration and experimentation (50, 58). This finding underscores the urgency of introducing comprehensive sexuality education at an early age (59). One of the findings that stood out especially was the subgroup analysis of school-based and community-based studies. The cases of sexual coercion were reported more significantly in school-based settings, and it can be assumed that schools are not only the learning areas but also the locations where the risks of sexual coercion may occur. While this is not surprising, it points to possibilities of cultural and institutional factors on reporting sexual coercion. In-school adolescents may feel empowered to speak unlike in studies conducted in communities where cultural factors, fear of reprimand or shame may influence reporting. Beyond its statistical relevance, the high rate of sexual coercion reported in schools underscores an urgent need for strategic engagement by the Ministry of Education, school health programmes, and youth-serving institutions to address the underlying issues promoting sexual coercion in the school environment. Sexual coercion can be prevented in schools by incorporating sexuality education in school curricula, peer mentorship programs, and training teachers on sexual issues and how to report such situations to prevent them. Also, school-based reporting systems, counseling, and referral channels connected to health services that are youth and adolescent friendly may offer immediate help to the survivors. Dealing with coercion in schools would hence involve multi-sectoral approach that incorporates teachers and parents, community leaders and health workers in the process which will result in a learning environment that is not only safe but also empowering.

We also observed an inverse relationship between prevalence and sample size, with smaller studies reporting higher estimates. This pattern is common in prevalence research and may reflect selection bias, sampling methods, or study setting differences. It emphasizes the importance of large-scale, nationally representative surveys to provide more precise and generalizable prevalence estimates.

The high level of association between sexual relationship and higher risk of coercion observed is consistent with past findings in Sub-Saharan Africa. For example, a studies from Ghana reported that an being a girlfriend and engaged in intimate relationship was a major risk condition when it comes to experiencing forced sex among youthful females (14). Likewise, a study conducted in Kenya established that in most cases, coercion was done in an intimate relationship as opposed to strangers (27). The reasoning is that being in a relationship exposes people to more sex and power-differentiations which makes them susceptible to coercive relationships. In fact, the odds ratio of this meta-analysis is found to be increased 4 times which is relatively large and indicates a significant mark to draw prevention initiatives. Further, this subgroup showed zero observed heterogeneity (*I*^2^ = 0) in the meta-analysis, which highlights the similarity of this risk factor in different study settings—which and once again, proves that sexual relationship status is a relatively stable predictor of coercion in these populations.

### Implication and contribution statement

The review has shown that sexual coercion continues to be a severe menace to the safety and wellbeing of African adolescents and young adults, and the school has become a potential high-risk setting. This observation requires an immediate cooperation between Ministries of Education and Health to ensure that schools are safe, conducive, and secure areas. There should be policies that require age-related sexuality and consent education, training of teachers and counselors, as well as confidential reporting and referral procedures. These policies must institutionalize age-appropriate sexuality and consent education, as recommended by the World Health Organization (60) and the United Nations Educational, Scientific and Cultural Organization (61). Additionally, training teachers, school counselors, and peer educators to detect, prevent, and respond to sexual coercion is crucial. Establishing confidential reporting and referral systems and linking schools to adolescent-friendly health services, as emphasized in the WHO Global Accelerated Action for the Health of Adolescents (AA-HA!) framework, will facilitate early detection, timely response, and psychosocial support for survivors (62).

Linking schools and health services to adolescents will also assist in early detection and support of the survivors. It is also significant to have a more multi-sectoral approach. Societies, parents, and religious organisations should be involved to challenge harmful gender standards and promote respect and consent. The structural factors like poverty, inequalities, and disparity in powers must be mitigated under specific social and economic initiatives. Improvement of community-based and school-based programs would result in safer learning environments and SDGs goals on gender equality, education, and health.

One of the major limitation of this meta-analysis is the fact that the degree of heterogeneity is high due to the fact that the overall effect sizes are widely different and cannot be attributed to chance only. Such heterogeneity is probably due to differences in the populations of studies, their setting, and, most significantly, definitional inconsistencies across studies. This heterogeneity has the implication that the estimate of the pooled effect might not be a true representation of a single homogeneous underlying association, but a representation of an average of contextually different phenomena. The definitional inconsistencies measurement made cross-country comparisons more challenging. Lack of data from North Africa also limits generalisability of this finding to that setting. Nevertheless, our study included data from 14 countries with over 60,000 participants, and the sub-group analyses highlighted important gender and regional differences that will inform development of context-specific policies and interventions.

This paper shows that there is a pressing need of a standardized, validated instrument of measuring sexual coercion. It would be better to define and adopt a short, similar definition to increase comparability among the studies, improve the validity of future meta-analyses, and ultimately provide more reliable information to inform the design of interventions and policy reactions to sexual coercion among adolescents and young adults.

## Conclusion

Sexual coercion is a major issue of public health and human right of concern among African adolescents and young adults, especially on female gender and school-going children. Ministries of health, education, and youth development need to focus on the school- and community-based programs that combine comprehensive sexuality education, focusing on consent, negotiation skills, and gender-equitable views on sex. The intervention must be gender and culturally sensitive, which involves parents, teachers, religious leaders and peers to transform unhealthy norms that uphold coercion and silence.

In order to further enhance the policy responses, government and research institutions should also invest in large scale, representative studies which cover the areas that are underrepresented like the Central Africa and rural populations, where the data is not abundant but the vulnerability is also high. A more consistent tool of measuring sexual coercion in different contexts would enhance comparability and temporal tracking. The national approaches to preventing sexual coercion should be framed in terms of a rights-based approach, where the rights of adolescents are acknowledged (their right to bodily autonomy, safety, and informed choice), which guarantees that all young people, irrespective of their gender and place of residence, should not be subjected to coercion and be provided with the opportunity to make the choice in terms of sex on the basis of informed consent.

## Data Availability

The datasets presented in this study can be found in online repositories. The names of the repository/repositories and accession number(s) can be found in the article/Supplementary Material.
