# Supplementary material for: Prevalence of sexual coercion and associated factors among adolescents and young adults in Africa: a systematic review and meta-analysis
Source: Front Reprod Health. 2025 Nov 28;7:1697868. doi: 10.3389/frph.2025.1697868 (PMC12698548; doi:10.3389/frph.2025.1697868)
Supplement: Supplementary file 1 [file Datasheet1.zip › Sexual Coercion_APPENDICES/APPENDIX B.pdf]

Appendix 1: Search Strategy Protocol

Source	Search Strategy	Result
**PubMed**	(("prevalence"[MeSH Terms] OR "prevalence"[Title/Abstract] OR "frequenc*"[Title/Abstract] OR "occurrence*"[Title/Abstract] OR "pattern*"[Title/Abstract]) AND ("prevalence"[MeSH Terms] OR "prevalence"[Title/Abstract] OR "frequenc*"[Title/Abstract] OR "occurrence*"[Title/Abstract] OR "pattern*"[Title/Abstract]) AND ("Sexual coercion"[Title/Abstract] OR "sexual violence"[Title/Abstract] OR "sexual assault"[Title/Abstract] OR "sexual abuse"[Title/Abstract] OR "non-consensual sex"[Title/Abstract] OR "sexual manipulation"[Title/Abstract] OR "sexual exploitation"[Title/Abstract]) AND ("adolescent"[MeSH Terms] OR "adolescen*"[Title/Abstract] OR "youth*"[Title/Abstract] OR "teen*"[Title/Abstract] OR "young people"[Title/Abstract] OR "young adult*"[Title/Abstract]) AND ("Algeria"[Title/Abstract] OR "Angola"[Title/Abstract] OR "Benin"[Title/Abstract] OR "Botswana"[Title/Abstract] OR "Burkina Faso"[Title/Abstract] OR "Burundi"[Title/Abstract] OR "Cabo Verde"[Title/Abstract] OR "Cameroon"[Title/Abstract] OR "Central African Republic"[Title/Abstract] OR "Chad"[Title/Abstract] OR "Comoros Islands"[Title/Abstract] OR "Congo Democratic Republic of"[Title/Abstract] OR "Congo Republic"[Title/Abstract] OR "Cote d Ivoire"[Title/Abstract] OR "Djibouti"[Title/Abstract] OR "Egypt"[Title/Abstract] OR "Equatorial Guinea"[Title/Abstract] OR "Eritrea"[Title/Abstract] OR "Eswatini"[Title/Abstract] OR "Ethiopia"[Title/Abstract] OR "Gabon"[Title/Abstract] OR "Gambia"[Title/Abstract] OR "Ghana"[Title/Abstract] OR "Guinea"[Title/Abstract] OR "Guinea-Bissau"[Title/Abstract] OR "Kenya"[Title/Abstract] OR "Lesotho"[Title/Abstract] OR "Liberia"[Title/Abstract] OR "Libya"[Title/Abstract] OR "Madagascar"[Title/Abstract] OR "Malawi"[Title/Abstract] OR "Mali"[Title/Abstract] OR "Mauritania"[Title/Abstract] OR "Mauritius"[Title/Abstract] OR "Morocco"[Title/Abstract] OR "Mozambique"[Title/Abstract] OR "Namibia"[Title/Abstract] OR "Niger"[Title/Abstract] OR "Nigeria"[Title/Abstract] OR "Rwanda"[Title/Abstract] OR "Sao Tome and Principe"[Title/Abstract] OR "Senegal"[Title/Abstract] OR "Seychelles"[Title/Abstract] OR "Sierra Leone"[Title/Abstract] OR "Somalia"[Title/Abstract] OR "South Africa"[Title/Abstract] OR "South Sudan"[Title/Abstract] OR "Sudan"[Title/Abstract] OR "Tanzania"[Title/Abstract] OR "Togo"[Title/Abstract] OR "Tunisia"[Title/Abstract] OR "Uganda"[Title/Abstract] OR "Zambia"[Title/Abstract] OR "Zimbabwe"[Title/Abstract])) AND (2000:2025[pdat])	494
**CINAHL**	XB (prevalence OR frequenc* OR occurrence* OR pattern*) AND XB (“Sexual coercion” OR “sexual violence” OR “sexual assault” OR “sexual abuse” OR “non-consensual sex” OR “sexual manipulation” OR “sexual exploitation”) AND XB (Adolescen* OR Youth* OR Teen* OR “young people” OR “young adult*”) AND XB (Algeria OR Angola OR Benin OR Botswana OR "Burkina Faso" OR Burundi OR Cameroon OR "Cape Verde" OR "Central African Republic" OR Chad OR Comoros OR Congo OR "Democratic Republic of the Congo" OR Djibouti OR Egypt OR "Equatorial Guinea" OR Eritrea OR Eswatini OR Ethiopia OR Gabon OR Gambia OR Ghana OR Guinea OR "Guinea-	96

	Bissau" OR Ivory Coast OR "Côte d’Ivoire" OR Kenya OR Lesotho OR Liberia OR Libya OR Madagascar OR Malawi OR Mali OR Mauritania OR Mauritius OR Morocco OR Mozambique OR Namibia OR Niger OR Nigeria OR Rwanda OR "São Tomé and Príncipe" OR Senegal OR Seychelles OR "Sierra Leone" OR Somalia OR "South Africa" OR "South Sudan" OR Sudan OR Tanzania OR Togo OR Tunisia OR Uganda OR Zambia OR Zimbabwe)	
**Scopus**	(TITLE-ABS-KEY (prevalence OR frequenc* OR occurrence* OR pattern*) AND TITLE-ABS-KEY ("Sexual coercion" OR "sexual violence" OR "sexual assault" OR "sexual abuse" OR "non-consensual sex" OR "sexual manipulation" OR "sexual exploitation") AND TITLE-ABS-KEY (Adolescen* OR Youth* OR Teen* OR "young people" OR "young adult*")) AND PUBYEAR > 1999 AND PUBYEAR < 2026 AND (LIMIT-TO (DOCTYPE , "ar")) AND (LIMIT-TO (AFFILCOUNTRY , "Algeria") OR LIMIT-TO (AFFILCOUNTRY , "Angola") OR LIMIT-TO (AFFILCOUNTRY , "Benin") OR LIMIT-TO (AFFILCOUNTRY , "Botswana") OR LIMIT-TO (AFFILCOUNTRY , "Burkina Faso") OR LIMIT-TO (AFFILCOUNTRY , "Burundi") OR LIMIT-TO (AFFILCOUNTRY , "Cameroon") OR LIMIT-TO (AFFILCOUNTRY , "Central African Republic") OR LIMIT-TO (AFFILCOUNTRY , "Chad") OR LIMIT-TO (AFFILCOUNTRY , "Congo") OR LIMIT-TO (AFFILCOUNTRY , "Cote d'Ivoire") OR LIMIT-TO (AFFILCOUNTRY , "Democratic Republic Congo") OR LIMIT-TO (AFFILCOUNTRY , "Egypt") OR LIMIT-TO (AFFILCOUNTRY , "Eritrea") OR LIMIT-TO (AFFILCOUNTRY , "Ethiopia") OR LIMIT-TO (AFFILCOUNTRY , "Gabon") OR LIMIT-TO (AFFILCOUNTRY , "Gambia") OR LIMIT-TO (AFFILCOUNTRY , "Ghana") OR LIMIT-TO (AFFILCOUNTRY , "Guinea") OR LIMIT-TO (AFFILCOUNTRY , "Guinea-Bissau") OR LIMIT-TO (AFFILCOUNTRY , "Kenya") OR LIMIT-TO (AFFILCOUNTRY , "Lesotho") OR LIMIT-TO (AFFILCOUNTRY , "Liberia") OR LIMIT-TO (AFFILCOUNTRY , "Madagascar") OR LIMIT-TO (AFFILCOUNTRY , "Malawi") OR LIMIT-TO (AFFILCOUNTRY , "Mali") OR LIMIT-TO (AFFILCOUNTRY , "Mauritania") OR LIMIT-TO (AFFILCOUNTRY , "Morocco") OR LIMIT-TO (AFFILCOUNTRY , "Mozambique") OR LIMIT-TO (AFFILCOUNTRY , "Namibia") OR LIMIT-TO (AFFILCOUNTRY , "Nigeria") OR LIMIT-TO (AFFILCOUNTRY , "Rwanda") OR LIMIT-TO (AFFILCOUNTRY , "Senegal") OR LIMIT-TO (AFFILCOUNTRY , "Sierra Leone") OR LIMIT-TO (AFFILCOUNTRY , "Somalia") OR LIMIT-TO (AFFILCOUNTRY , "South Africa") OR LIMIT-TO (AFFILCOUNTRY , "South Sudan") OR LIMIT-TO (AFFILCOUNTRY , "Sudan") OR LIMIT-TO (AFFILCOUNTRY , "Swaziland") OR LIMIT-TO (AFFILCOUNTRY , "Tanzania") OR LIMIT-TO (AFFILCOUNTRY , "Togo") OR LIMIT-TO (AFFILCOUNTRY , "Tunisia") OR LIMIT-TO (AFFILCOUNTRY , "Uganda") OR LIMIT-TO (AFFILCOUNTRY , "Zambia") OR LIMIT-TO (AFFILCOUNTRY , "Zimbabwe"))	794
**WebOfScience**	(((TS=(prevalence)) OR TS=(frequenc*)) OR TS=(occurrence*)) OR TS=(pattern*) AND ((((((TS=(“Sexual coercion”)) OR TS=(“sexual violence”)) OR TS=(“sexual assault”)) OR TS=(“sexual abuse”)) OR TS=(“non-consensual sex”)) OR TS=(“sexual manipulation”)) OR TS=(“sexual exploitation”) AND ((((TS=(Adolescen*)) OR TS=(Youth*)) OR TS=(Teen*)) OR TS=(“young people”)) OR TS=(“young adult*”) AND 2025 or 2024 or 2023 or 2022 or 2020 or 2021 or 2019 or 2018 or 2017 or 2016 or 2015 or 2014 or 2013 or 2012 or 2011 or 2010 or 2009 or 2008 or 2007 or 2006 or 2005 or 2004 or 2003 or 2002 or 2001 or 2000 (Publication Years) AND Article (Document Types) AND Algeria OR Angola OR Benin OR Botswana OR "Burkina Faso" OR Burundi OR Cameroon OR "Cape Verde" OR	424

	"Central African Republic" OR Chad OR Comoros OR Congo OR "Democratic Republic of the Congo" OR Djibouti OR Egypt OR "Equatorial Guinea" OR Eritrea OR Eswatini OR Ethiopia OR Gabon OR Gambia OR Ghana OR Guinea OR "Guinea-Bissau" OR Ivory Coast OR "Côte d'Ivoire" OR Kenya OR Lesotho OR Liberia OR Libya OR Madagascar OR Malawi OR Mali OR Mauritania OR Mauritius OR Morocco OR Mozambique OR Namibia OR Niger OR Nigeria OR Rwanda OR "São Tomé and Príncipe" OR Senegal OR Seychelles OR "Sierra Leone" OR Somalia OR "South Africa" OR "South Sudan" OR Sudan OR Tanzania OR Togo OR Tunisia OR Uganda OR Zambia OR Zimbabwe (Countries/Regions)	
