# Supplementary material for: Prevalence of sexual coercion and associated factors among adolescents and young adults in Africa: a systematic review and meta-analysis
Source: Front Reprod Health. 2025 Nov 28;7:1697868. doi: 10.3389/frph.2025.1697868 (PMC12698548; doi:10.3389/frph.2025.1697868)
Supplement: Supplementary file 1 [file Datasheet1.zip › Sexual Coercion_APPENDICES/APPENDIX C.pdf]

Component	Inclusion Criteria	Exclusion Criteria
Population (P)	Adolescents and young adults aged 10–24 years residing in African countries (both in-school and out-of-school). Studies with disaggregated data for this age group were eligible.	Studies outside Africa, populations not within the 10–24 age range, or those without disaggregated data.
Exposure (E)	Experience of sexual coercion, defined as non-consensual sexual activity resulting from psychological, emotional, social, or economic pressure, manipulation, intimidation, or exploitation (including penetrative and non-penetrative forms)	Studies not specifically addressing sexual coercion, or those combining sexual coercion with other forms of violence without separate reporting.
Outcome (O)	Reported prevalence of sexual coercion, with or without associated demographic, social, or behavioral factors.	Studies not reporting prevalence of sexual coercion.
Study Design (S)	Quantitative observational studies (cross-sectional, baseline cohort data, and mixed-methods with extractable quantitative prevalence data).	Qualitative studies, intervention studies, case reports, case series, reviews, editorials, or commentaries.
