# Supplementary material for: Prevalence of sexual coercion and associated factors among adolescents and young adults in Africa: a systematic review and meta-analysis
Source: Front Reprod Health. 2025 Nov 28;7:1697868. doi: 10.3389/frph.2025.1697868 (PMC12698548; doi:10.3389/frph.2025.1697868)
Supplement: Supplementary file 1 [file Datasheet1.zip › Sexual Coercion_APPENDICES/APPENDIX G.pdf]

Sensitivity Test of Included Studies

Study Excluded	Pooled Prevalence (95% CI)	I² (%)	Change	Interpretation
None (All Included)	0.20 [0.17, 0.23]	99%	-	Baseline
Amo-Adjei et al., 2025[23]	0.20 [0.17, 0.23]	99%	0	No change
Bekele et al., 2011[24]	0.20 [0.16, 0.23]	99%	0	No change
Beyene et al., 2020[25]	0.20 [0.17, 0.23]	99%	0	No change
Chime et al., 2021[26]	0.19 [0.16, 0.23]	99%	-0.01	Negligible change
Decraen et al., 2012[27]	0.20 [0.17, 0.23]	99%	0	No change
Fan et al., 2016[29]	0.19 [0.16, 0.23]	99%	-0.01	Negligible change
Garoma et al., 2008[30]	0.20 [0.16, 0.23]	99%	0	No change
Garoma Abeya, 2022[31]	0.19 [0.16, 0.23]	99%	-0.01	Negligible change
Goessmann et al., 2020[32]	0.20 [0.17, 0.23]	99%	0	No change
Haile et al., 2013[33]	0.20 [0.17, 0.23]	99%	0	No change
Landis et al., 2018[34]	0.20 [0.17, 0.23]	99%	0	No change
Maharaj & Munthree, 2007[55]	0.19 [0.16, 0.22]	99%	0	No change
Meinck et al., 2016[36]	0.20 [0.17, 0.24]	99%	0	No change
Mlyakado & Li, 2019[37]	0.20 [0.17, 0.23]	99%	0	No change
Moore et al., 2012[38]	0.20 [0.17, 0.24]	99%	0	No change
Moore et al., 2012[38]	0.20 [0.17, 0.23]	99%	0	No change
Moore et al., 2012[38]	0.20 [0.17, 0.24]	99%	0	No change

Moore et al., 2012[38]	0.20 [0.17, 0.24]	99%	0	No change
Naidoo et al., 2017[39]	0.20 [0.17, 0.23]	99%	0	No change
Nguyen et al., 2019[40]	0.20 [0.17, 0.23]	99%	0	No change
Nguyen et al., 2019[40]	0.20 [0.17, 0.24]	99%	0	No change
Nguyen et al., 2019[40]	0.20 [0.17, 0.23]	99%	0	No change
Odeyemi et al., 2016[41]	0.20 [0.17, 0.24]	99%	0	No change
Owusu-Addo et al., 2023[42]	0.20 [0.17, 0.24]	99%	0	No change
Perry et al., 2020[43]	0.20 [0.17, 0.23]	99%	0	No change
Richter et al., 2018[4]	0.20 [0.17, 0.23]	99%	0	No change
Rizo et al., 2021[44]	0.19 [0.16, 0.22]	99%	-0.01	Negligible change
Rumble et al., 2015[45]	0.20 [0.17, 0.24]	99%	0	No change
Seidu et al., 2024[5]	0.18 [0.16, 0.21]	99%	-0.02	Negligible change
Stark et al., 2017[46]	0.20 [0.17, 0.23]	99%	0	No change
Stark et al., 2017[46]	0.20 [0.16, 0.23]	99%	0	No change
Swedo et al., 2019[47]	0.19 [0.16, 0.22]	99%	-0.01	Negligible change
Tenkorang et al., 2021[48]	0.20 [0.17, 0.23]	99%	0	No change
Tusiime et al., 2015[49]	0.20 [0.17, 0.23]	99%	0	No change
Ybarra et al., 2012[51]	0.19 [0.16, 0.23]	99%	-0.01	Negligible change
Zablotska et al., 2009[52]	0.20 [0.17, 0.23]	99%	0	No change
